# The DNA binding domain and the C-terminal region of DNA Ligase IV specify its role in V(D)J recombination

**DOI:** 10.1371/journal.pone.0282236

**Published:** 2023-02-24

**Authors:** Vidyasagar Malashetty, Audrey Au, Jose Chavez, Mary Hanna, Jennifer Chu, Jesse Penna, Patricia Cortes

**Affiliations:** 1 Immunology Institute, Icahn School of Medicine at Mount Sinai, New York, NY, United States of America; 2 Department of Molecular, Cellular and Biomedical Sciences, City University of New York School of Medicine, City College of New York, New York, NY, United States of America; Tulane University Health Sciences Center, UNITED STATES

## Abstract

DNA Ligase IV is responsible for the repair of DNA double-strand breaks (DSB), including DSBs that are generated during V(D)J recombination. Like other DNA ligases, Ligase IV contains a catalytic core with three subdomains—the DNA binding (DBD), the nucleotidyltransferase (NTD), and the oligonucleotide/oligosaccharide-fold subdomain (OBD). Ligase IV also has a unique C-terminal region that includes two BRCT domains, a nuclear localization signal sequence and a stretch of amino acid that participate in its interaction with XRCC4. Out of the three mammalian ligases, Ligase IV is the only ligase that participates in and is required for V(D)J recombination. Identification of the minimal domains within DNA Ligase IV that contribute to V(D)J recombination has remained unresolved. The interaction of the Ligase IV DNA binding domain with Artemis, and the interaction of its C-terminal region with XRCC4, suggest that both of these regions that also interact with the Ku70/80 heterodimer are important and might be sufficient for mediating participation of DNA Ligase IV in V(D)J recombination. This hypothesis was investigated by generating chimeric ligase proteins by swapping domains, and testing their ability to rescue V(D)J recombination in Ligase IV-deficient cells. We demonstrate that a fusion protein containing Ligase I NTD and OBDs flanked by DNA Ligase IV DBD and C-terminal region is sufficient to support V(D)J recombination. This chimeric protein, which we named Ligase 37, complemented formation of coding and signal joints. Coding joints generated with Ligase 37 were shorter than those observed with wild type DNA Ligase IV. The shorter length was due to increased nucleotide deletions and decreased nucleotide insertions. Additionally, overexpression of Ligase 37 in a mouse pro-B cell line supported a shift towards shorter coding joints. Our findings demonstrate that the ability of DNA Ligase IV to participate in V(D)J recombination is in large part mediated by its DBD and C-terminal region.

## Introduction

The process of V(DJ) recombination that occurs during B and T cell development generates a diverse repertoire of B and T cell receptors, and it is essential for the formation of a functional adaptive immune system [1]. During V(D)J recombination, recombination signal sequences (RSSs), are recognized and cleaved by the recombination activating genes 1 and 2 (Rag1 and Rag2). Rag-mediated cleavage leads to the generation of two physically different double stranded DNA ends; a sealed hairpin coding end and a blunt 5’ phosphorylated signal end [2]. Processing and joining of the coding ends and joining of the signal ends are mediated by proteins of the non-homologous end joining (NHEJ) pathway [3]. The NHEJ factors Ku70/80, DNA-PKcs, Artemis, DNA Ligase IV (Lig-IV), XRCC4, and XLF/Cernunnos contribute to generate the final products of V(D)J recombination, the coding and signal joints; with Artemis and DNA-PKcs having a well-documented role in coding joint formation and a less clear and less characterized function during signal joint formation [4–8]. Additional factors, including pol mu, pol lambda and TdT contribute to increase junctional diversity [9].

Despite a similar domain organization between the three mammalian ligases, Lig-IV is the only ligase capable of participating in V(D)J recombination, and it is essential for the reaction [10, 11]. Published studies have also provided evidence for a central role of Lig-IV in regulating the processing of incompatible DNA ends and in promoting rapid ligation of double strand DNA ends once the ends become compatible [12]. In this context a structural role for Lig-IV that includes holding the DNA end together in a short-range complex, relevant for promoting end joining fidelity, was proposed [13]. Whether this or other models that include mechanistic flexibility at multiple steps of the DNA repair process [7, 14] function during V(D)J recombination, a reaction that requires the generation of diverse coding joints by changing and making new DNA sequences, remains an interesting and largely unanswered question.

Lig-IV, like other DNA ligases, has a core catalytic region composed of a DNA binding domain (DBD), the nucleotidyltransferase (NTD), and the oligonucleotide/oligosaccharide-fold subdomains (OBD) [15–18]. Lig-IV also has a C-terminal region that contains two BRCT domains, a nuclear localization signal sequence, and a stretch of amino acids located between the BRCT domains that participates in the interaction of Lig-IV with XRCC4 [17–20]. Lig-IV C-terminal region, specifically the N-terminal BRCT domain, interacts with Ku in a direct protein-protein interaction that might contribute to the stability of the repair complexes [21]. In addition, the C-terminal region of Lig-IV is required for its efficient localization to the nucleus, also influencing the nuclear localization of XRCC4 [22]. On the other hand, Lig-IV DBD interacts specifically with the C-terminal region of Artemis [15, 23, 24], the nuclease that opens hairpin coding ends during V(D)J recombination [25]. In addition to interacting with Artemis and DNA [23, 24], the DBD of Lig-IV was recently shown to interact with Ku70 [21], and to play a key role in guiding the processing of double stranded DNA ends [12, 26].

Based on our work and other publications, we hypothesized that the ability of Lig-IV to participate in V(D)J recombination, at a minimum, depends on its DBD and its C-terminal region. This hypothesis was tested by generating chimeric proteins that allowed the substitution of Lig-IV domains with DNA Ligase I (Lig-I) domains. Results presented in this study demonstrate that the DBD and C-terminal region of Lig-IV specify the role of Lig-IV in V(D)J recombination.

## Materials and methods

### Expression vectors

Generation of chimeric ligase constructs was carried out by PCR amplification of Lig-I and Lig-IV domains using a fusion PCR approach with the primers listed in Table 1. Final fusion products were cloned into the pEF-Flag expression vector using BamHI and NotI restriction sites. The BamHI site present in the human Ligase I cDNA was destroyed using oligo JP014 as the same restriction site was used for cloning. All PCR-generated chimeric ligases were verified by Sanger sequencing.

**Table 1:** PCR Primers used for generation of expression vectors.

Construct	Primer name	Primers used for each construct
Ligase I	JP012	5’-atgacgcggatcctcatctccaatgcagcgaagtatcatgtcatttttc-3 5’- atagtttagcggccgcttagtaggtatcttcagggtcagagcctga-3’ 5’-atgacgcggatcctcatctccagatccatctggttacaatcctgcc-3’
Lig I-BamHI site mutated	JP013	
	JP014	
Ligase I core	L1Core-F	5’-cgcggatccgatccatctggttacaat3’ 5’atagtttagcggccgcttagtaggtatcttcagggtcagagcctga-3
	JP013	
Ligase IV	LigIVBamHI-F	5’-ctgggatccatggctgcctcacaaacttcac-3’ 5’- atagtttagcggccgcttaaatcaaatactggttttcttc-3’
	LigIV911NotI-R	
LigaseIV core	LigIVBamHI-F	5’-ctgggatccatggctgcctcacaaacttcac-3 5’-atagtttagcggccgctttttaaccacctatataaag-3’
	LigIV1core-R	
Ligase 36	L1Core-F	5’-cgcggatccgatccatctggttacaat-3’ 5’-gaccctgaagatacctacggtaagctcgcatctaaa-3’ 5’-tttagatgcgagcttaccgtaggtatcttcagggtc-3’
	L1(919)-L4(608)-F	
	L1(919)-L4(608)-R	
	JP017	5’-atagtttagcggccgcttaaatcaaatactggttttcttcttgtaattcac-3’
Ligase 37	LigIVBamHI-F L4(245)-L1 (535)-F L4(245)-L1 (535)-R L1(919)-L4(608)-F L1(919)-L4(608)-R JP017	5’-ctgggatccatggctgcctcacaaacttcac-3’ 5’-atcactttattttctgcaagcccagggattcccctg-3’ 5’-caggggaatccctgggcttgcagaaaataaagtgat-3’ 5’-gaccctgaagatacctacggtaagctcgcatctaaa-3’ 5’-tttagatgcgagcttaccgtaggtatcttcagggtc-3’ 5’-atagtttagcggccgcttaaatcaaatactggttttcttcttgtaattcac-3’
Ligase 43	L1Core-F	5’-cgcggatccgatccatctggttacaat-3’ 5’-atagtttagcggccgcttagctcagcttgcagtgctccgg-3’ 5’-atcactttattttctgcaagcccagggattcccctg-3’ 5’-atagtttagcggccgcttaaatcaaatactggttttcttcttgtaattcac-3’
	JP015	
	L4(245)-L1 (535)-F	
	JP017	

Three chimeric ligases were generated. Ligase 36 (Lig-36) contains the catalytic core of Lig-I (DBD, NTD, and OBD) and the C-terminal region of Lig-IV, Ligase 37 (Lig-37) with the DBD and C-terminal region from Lig-IV fused to the NTD and OBD of Lig-I, and Ligase 43 (Lig-43) with the DBD of Lig-I fused to the NTD, OBD, and C-terminal region of Lig-IV. To generate retroviral vectors for transduction of Lig-IV-deficient cells, control and chimeric ligases from pEF-Flag ligase constructs were subcloned into the retroviral vector pRETRO-MCS-IRES-Blasticidin, as described by Francis et al., 2014 [22]. Lig-I cDNA was obtained from the laboratory of Dr. Tomas Lindahl (Cancer Research UK London Research Institute) [27].

### Cell lines and culture conditions

Human pre-B cell lines Nalm 6 and the Lig-IV-deficient N114P2 [11] were obtained from Dr. Michael Lieber (University of Southern California, Los Angeles, CA), mouse Rag2 -/- pro-B cells [28] and Lig-IV-deficient mouse embryonic fibroblasts (MEFs) [29] were obtained from Dr. Fred Alt (Harvard Medical School, Boston, MA). Growth medium and conditions for human pre-B cells, mouse pro-B cells and MEFs were as described previously [22, 30]. Briefly, human pre-B cells and mouse pro-B cells were cultured in RPMI (Gibco), 10% heat inactivated Fetal Bovine Serum (Hyclone), 1% antibiotic–antimycotic, 1 mM sodium pyruvate, 2 mM glutamine (Cellgro), and 100 μM β-mercaptoethanol (β-ME) (Sigma). Stable cell lines transduced with ligase expressing constructs were selected and maintained with 10 μg/ml Blasticidin. MEFs were cultured on plates pretreated with 0.2% gelatin in DMEM, 10% heat inactivated Fetal Bovine Serum (Invitrogen), 1% antibiotic-antimycotic, 1 mM sodium pyruvate, 2 mM glutamate, 1% MEM non-essential amino acids (Cellgro), and 20 mM HEPES (Cellgro). Cells were cultured at 37°C and 5% CO_2_.

### Antibodies

Rabbit polyclonal antibodies against Lig-IV amino acids (aa) 1–240 were generated in our laboratory [23]. The anti-XRCC4 antibody was purchased from Serotec. Mouse monoclonal antibodies against the Flag-peptide and Actin were from Sigma. Secondary antibodies HRP conjugated anti-mouse, and anti-rabbit IgG are from Thermo Scientific.

### Expression and affinity purification of Flag-tagged ligases

Expression of chimeric and control ligases was done by transient transfection, via electroporation of Lig-IV-deficient human pre-B cells (N114P2). 48 hours after electroporation, extracts were prepared and used for affinity purification using Flag beads followed by elution with the Flag peptide. To ensure comparable protein expression of the different ligases, expression vectors were first titrated. Protein levels of affinity purified Flag-tagged ligase were analyzed via western blot. Based on the titration experiments the following amounts of expression vectors were transfected 50 ng of Lig-I, 50 ng Lig-I core, 3 μg of Lig-IV, 2.5 μg of Lig-36, 7 μg of Lig-37, 20 μg Lig-43 and 20 μg of Lig-IV core. The total amount of transfected DNA was adjusted to 20 μg using the empty expression vector pEF. For each transfection, 2x10^7^ healthy growing N114P2 cells (at a density of 1.5x10^6^ cell/ml) were washed with plain RPMI, and resuspended in 200 μl of plain RPMI before transfection. DNA was added to the cells in a maximum volume of 10 μl. Cells and DNA were mixed and transferred to a 0.2μm electroporation cuvette. Cells were electroporated with a BioRad Gene Pulser II. The system was set at 230V and 975μF. Resistance was set at infinity. Electroporated cells were transferred to 100 mm dishes containing 10 ml of complete RPMI and cultured for 48 hours before they were harvested for preparation of TCE and Flag affinity purification. Cell extracts from Lig-IV-deficient N114P2 cells expressing Flag-tagged ligase constructs were prepared in Buffer A (25 mM Tris–HCl pH 8.0, 150 mM KCl, 10% glycerol, 0.1% Triton X-100, 0.5 mM EDTA, 1 mM DTT) with protease inhibitors (HALT Protease Inhibitor Cocktail Mix and 0.1 mM PMSF). Extracts were sonicated and incubated with 200 μg/ml Ethidium bromide at 4°C for 30 min and centrifuged at 35,000 rpm for 45 min. The supernatant was used for Flag immunopurification with anti-Flag M2-agarose beads (Sigma). Beads were washed multiple times with Buffer C (25 mM Tris-HCl pH 8.0, 150 mM KCl, 20% glycerol). Bound proteins were eluted with 0.1 mg/ml Flag peptide (Sigma) in Buffer C. Eluted proteins were used for both western blot analysis and ligation assays.

### Double-stranded DNA ligation assay

*In vitro* ligation assay was performed as described by Riballo et al., 2001 [31] with modifications as follows. Flag affinity purified ligases containing comparable amount of protein (based on anti-Flag western blot analysis) were incubated for 2 hours at 37°C in a final volume of 30 μl. The reaction mix included a 446 bp *Afl*III-*Pst*I ^32^P labeled fragment (with 5’ and 3’ non-compatible overhang ends) from the Bluescript plasmid, 50 mM triethanolamine, pH 7.5, 2 mM Mg(OAc)_2,_ 2 mM dithiothreitol, 0.1 mg/ml bovine serum albumin, 12% polyethylene glycol, and 0.2 mM ATP. The final salt concentration was adjusted to 75 mM KCl using buffer A. After incubation, reactions were stopped with 10 μl of stop mix (10 mM Tris pH7.5, 1 mM EDTA and 0.1% SDS) and 2 μl proteinase K and incubated for 1 hour at 50°C. Samples were subsequently phenol/chloroform-extracted and precipitated. Reaction products were resolved on 1.5% agarose gels. Dried gels were visualized using a Typhoon Phosphor Imager (GE).

### Recombination assay in Lig-IV-deficient cells

Lig-IV-deficient N114P2 human pre-B cells (2×10^7^) were electroporated with 1 μg pGG51 (coding joint reporter) or pGG49 (signal joint reporter) plasmids [32] and with one of the following expression vectors: Flag-pEF expression vectors (50 ng of Lig-I, 50 ng Lig-I core, 3 μg of Lig-IV, 2.5 μg of Lig-36, 7 μg of Lig-37, 20 μg Lig-43 and 20 μg of Lig-IV core) in each reaction. The amount of DNA used for the different ligases was pre-determined to ensure comparable protein expression among the different vectors. Electroporated cells were allowed to grow for 48 hours post-transfection to enable V(D)J recombination. V(D)J recombined plasmids were recovered from transfected cells using a plasmid mini-prep kit. The recovered plasmids were then subjected to *Dpn*I restriction enzyme digestion to remove non-replicated plasmids and potentially un-transfected substrates. This DNA was then used to transform DH5α *E*. *coli*, which were plated in parallel on ampicillin (Amp plates;100 μg/ml) and ampicillin plus chloramphenicol (Amp/Cam plates; 100 μg/ml and 34 μg/ml, respectively) plates. The Amp/Cam plates specifically select for colonies transformed with the recombined plasmid, which gains chloramphenicol resistance after V(D)J recombination [33]. Colonies formed on the Amp plates measure the transformation efficiency. Recombination frequency was calculated by dividing the total number of Amp/Cam colonies by the total number of Amp colonies and multiplying the resulting value by 100. To further analyze the coding junctions, we recovered rearranged pGG51 plasmid DNA from Amp/Cam-resistant colonies. Recovered recombined DNA was sequenced and analyzed for coding joint length, as well as nucleotide deletions and insertions. Three independent transfections, with three biological replicates each, were carried out for each experiment.

Analysis of signal joint formation in Lig-IV-deficient N114P2 cells was done as described above with the following modifications: the signal joint substrate pGG49 was used, and after calculating the signal joint frequency, recombined DNA was isolated from Amp/Cam colonies for signal joint analysis. Signal joints, when formed without modification, generate the ApaL1 restriction site (GTGCAC) that was used to evaluate the integrity of the signal joint generated by the different ligases. Signal joint analysis was performed in two independent experiments with three biological replicates each.

To complete the signal joint analysis in Lig-IV-deficient MEFs, cells were transfected by electroporation with the ligase expression vectors (0.2 μg of Lig-I, 0.2 μg of Lig-I core, 5 μg of Lig-IV, 3 μg of Lig-36, 7 μg of Lig-37, 20 μg of Lig-43, and 7 μg of Lig-IV core) along with Rag1 (2μg), Rag2 (2μg), and the signal joint recombination substrate pJH200 (1μg) [34]. The amount of ligase expression vectors was predetermined to ensure comparable protein expression, and the total amount of transfected DNA was adjusted to 25 μg with the empty expression pEF vector. Cells were harvested after 48 hours and plasmid DNA was purified and used to measure signal joint formation via the colony formation assay [34]. The integrity of the signal joints was analyzed by ApaL1 digestion as indicated above. Signal joint analysis in Lig-IV-deficient MEFs was done in two independent experiments with three biological replicates in each experiment.

### Endogenous recombination assay for DJ_H3_ junctional analysis

Mouse Rag2^-/-^ pro-B cells were transduced with Lig-IV or Lig-37 expressing retroviral vectors to generate stable cell lines. These Rag2-deficient mouse pro-B cells expressing endogenous as well as exogenous ligases were then transduced with Rag2 retrovirus to induce recombination. After 8 days in culture, genomic DNA (gDNA) was isolated and recombination was assessed. 50 μl PCR reactions using 200, 100, or 50 ng of template gDNA were done as published [30, 35, 36]. Primers used for this study have been described [35, 37]. DJ_H3_ PCR products were separated on a 1.5% agarose gel. A PCR-amplified RAG1 fragment from aa 589–758 served as control. The DJ_H3_ PCR products were cloned into pGEM-T Easy Vector (Promega), sequenced and analyzed for coding joint length.

## Results and discussion

### Generation and characterization of chimeric ligase constructs

**Fig 1:**
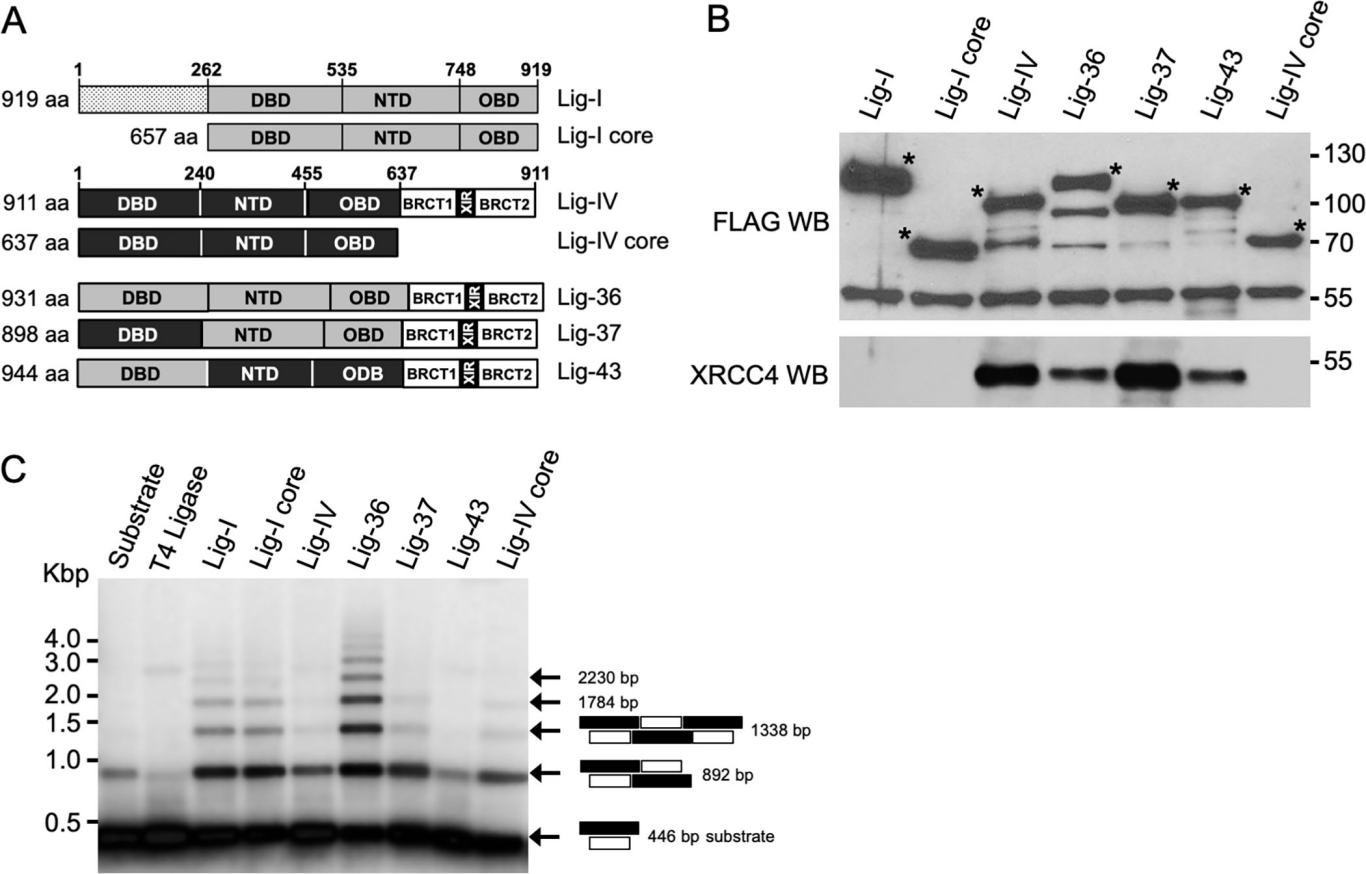
Expression, purification, XRCC4 interaction, and *in vitro* ligation activity of chimeric ligases. **A.** Schematic representation of Lig-I, Lig-IV and chimeric ligase domain constructs organization. Lig-I (1–919 aa) is a full-length Lig-I with its N-terminal region, DBD, NTD and OBD domains. Lig-I core is Lig-I without the N-terminal region. Lig-IV (1–911 aa) is a full-length Lig-IV with a DBD, NTD, OBD and its C-terminal region. Lig-IV core is Lig-IV without its C-terminal region. Lig-36 consists of the DBD, NTD and OBD of Lig-I and the C-terminal region of Lig-IV. Lig-37 consists of the NTD and OBD of Lig-I and the DBD and C-terminal region of Lig-IV. Lig-43 contains the DBD of Lig-I and the NTD, OBD and C-terminal region of Lig-IV. Each domain is depicted with numbers indicating the amino acid positions within the ligase. The total number of amino acids for each ligase is shown to the left of the schematic representation. **B.** Analysis of Flag-affinity purified ligases and their interaction with XRCC4. Western blot analysis of purified ligases using anti-Flag antibody (top panel). In each lane the asterisk indicates the full-length protein. The apparent molecular weight is as expected except for Lig-43 that migrates faster than expected, yet, it still shows a molecular weight that is higher than that of Lig-37. Interaction of chimeric ligases with XRCC4 was determined using an anti-XRCC4 antibody to detect XRCC4 in the affinity purified ligases (bottom panel). **C.** *In vitro* double strand DNA ligation activity of affinity purified chimeric ligases. A 446-bp double stranded DNA 5’ ^32^P-labeled substrate was incubated for 2 hours with the indicated ligase proteins. DNA was purified and separated in 1.5% agarose gel. The dried gel was analyzed on a phosphorimager. A schematic representation of the substrate (a 5’ ^32^P labeled 446 bp AflIII/PstI Bluescript fragment), and the potential ligation products are depicted toward the right of the figure. The reaction buffer with substrate alone served as the negative control for the ligation reaction.

To determine whether the Lig-IV DBD and C-terminal region were sufficient to allow participation of Lig-IV in V(D)J recombination, chimeric proteins were generated by domain swapping using protein domains from Lig-I and Lig-IV (Fig 1A). Three chimeric proteins were analyzed: Lig-36 that contains the catalytic core of Lig-I (DBD, NTD, and OBD) and the C-terminal region of Lig-IV, Lig-37 with the DBD and C-terminal region from Lig-IV fused to the NTD and OBD of Lig-I, and Lig-43 with the DBD of Lig-I fused to the NTD, OBD, and C-terminal region of Lig-IV. Lig-I, and Lig-I core, as well as Lig-IV and Lig-IV core were used as controls. To allow for affinity purification, all constructs were tagged at the N-terminal with a DNA sequence that encodes expression of the Flag tag. Plasmids and retroviral expression vectors were generated for all the Flag-tagged proteins depicted in Fig 1A.

Lig-IV-deficient human pre-B cells, N114P2, were transfected by electroporation with the expression vectors depicted in Fig 1A. Initial expression analysis indicated that the ligases were not all expressed at the same levels. Therefore, to obtain comparable protein expression levels before embarking in the comparative functional analysis of the chimeric proteins, all the vectors were titrated. These experiments indicated that comparable levels of protein expression could be obtained by transfecting 50 ng of Lig-I, 50 ng of Lig-I core, 3 μg of Lig-IV, 2.5 μg of Lig-36, 7 μg of Lig-37, 20 μg of Lig-43, and 20 μg of Lig-IV core. 48 hours after transfecting the indicated amounts of expression vectors, extracts were prepared and used for Flag-affinity purification of the Flag-tagged ligases. After elution with a Flag peptide, purified proteins were analyzed by western blot using anti-Flag antibodies (Fig 1B, top panel). As expected from the titration experiments, when transfected with the indicated amount of expression vectors, all ligases were expressed at comparable levels. Degradation products were present in the Lig-IV and Lig-36 samples however, the expected full-length products were predominant (indicated with an asterisk). Of note is that Lig-43 (944 amino acids) migrates faster than Lig-36 (931 amino acids), yet its molecular weight was still higher than that of Lig-37 (898 amino acids). This anomalous migration could be related to the amino acid composition. A band above the 55 kDa marker was observed in all lanes, this is likely to be a contamination with the heavy chain of the anti-Flag antibody that was used for immunopurification.

Anti-XRCC4 antibodies were used to investigate the interaction of the chimeric ligases with this NHEJ factor (Fig 1B, bottom panel). Lig-IV, Lig-36, Lig-37 and Lig-43 were able to interact with XRCC4. This suggests that the C-terminal region of Lig-IV present in Lig-36, Lig-37, and Lig-43 is properly folded and capable of engaging in protein-protein interactions. Interestingly, the interaction of XRCC4 with Lig-IV and Lig-37 was consistently higher across multiple experiments. As expected, Lig-I, Lig-I core and Lig-IV core did not interact with XRCC4 (Fig 1B, bottom panel).

The affinity purified Flag-tagged chimeric proteins (Fig 1B) were tested for *in vitro* ligation activity using a 5’ ^32^P labeled linear AflIII/PstI Bluescript fragment. This 446 bp fragment contains non-compatible 5’ and 3’ overhangs that are expected to minimize formation of circular monomers [31]. The substrate alone shows a contaminant product that migrates at the same position as a ligated dimer, making visualization of this ligated product less clear. Despite this technical problem we could observe that out of the three chimeric proteins, Lig-36 showed the highest level of *in vitro* ligation activity. The observed ligation activity for Lig-37 was low and comparable to the level observed for Lig-IV. Lig-43 on the other hand appears unable to ligate the substrate (Fig 1C). Together, data presented in Fig 1 indicates that the chimeric proteins are expressed, are able to interact with XRCC4, and that Lig-36 and Lig-37 have *in vitro* ligation activity.

### Lig-37 containing the Lig-IV DBD and C-terminal region supports coding joint formation

**Fig 2:**
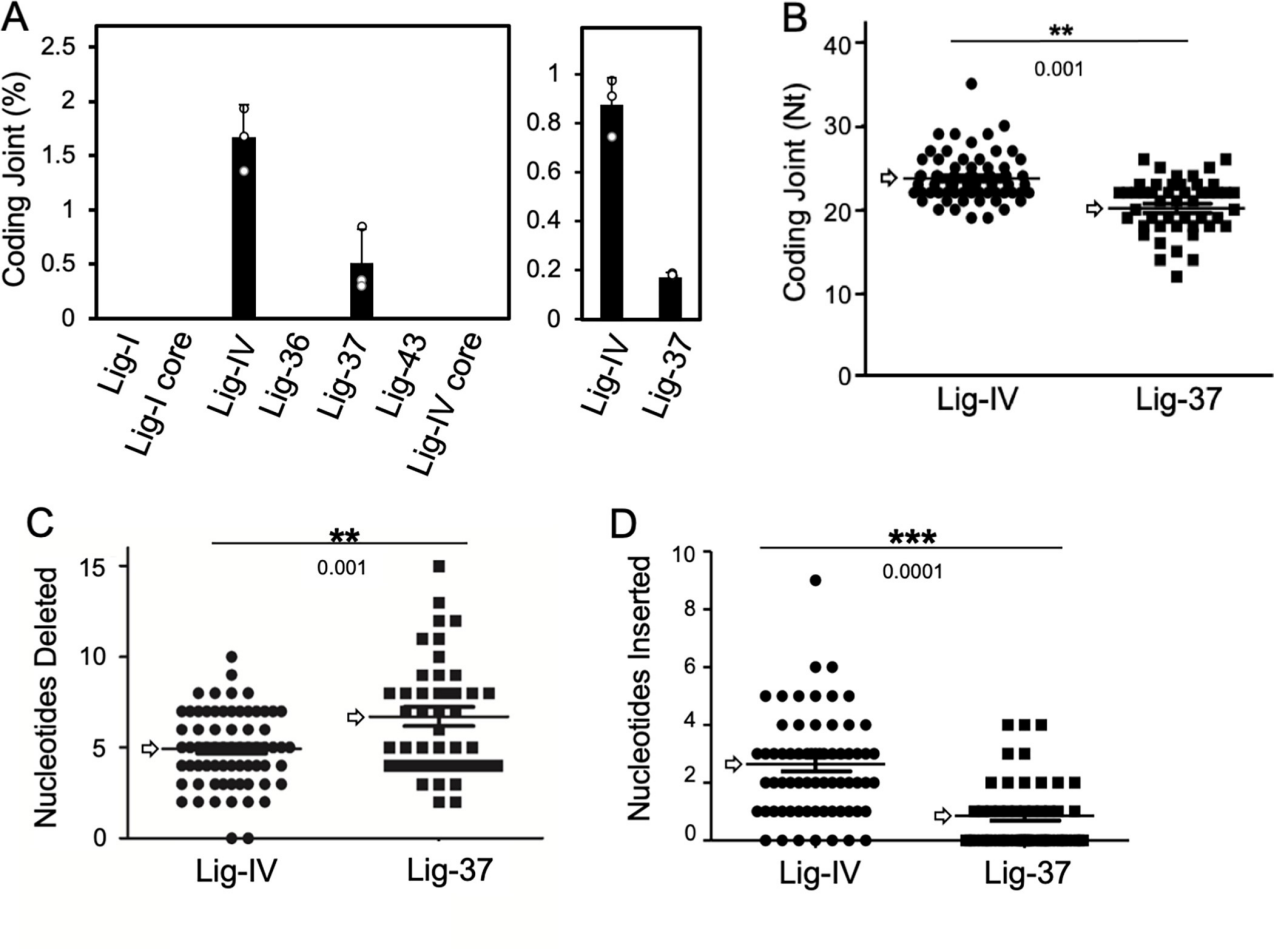
Chimeric protein Lig-37 supports formation of coding joints. **A.** Coding joint formation activity of chimeric ligases. Lig-IV-deficient human pre-B cells (N114P2) were transiently transfected with the following Flag-tagged expression vectors: Lig-I (50 ng), Lig-I core (50 ng), Lig-IV (3 μg), Lig-36 (2.5 μg), Lig-37 (7 μg), Lig-43 (20 μg) and Lig-IV core (20 μg), along with the coding joint (CJ) substrate, pGG51. Two days post transfection, plasmid DNA was recovered and digested with DpnI restriction enzyme before transformation. Coding joint formation was determined by a colony formation assay as described [32]. Percentage of coding joint formation is presented on the y-axis. Error bars are from the evaluation of triplicates, with independent data points also depicted within the graph (white dots). An independent coding joint formation assay for Lig-IV and Lig-37 done with three biological replicates each is also presented (left side). For all other ligases that show no activity, the result from one experiment done with three biological replicates for each ligase is shown. However, the complete set of ligases was analyzed three times with three biological replicates in each independent experiment. **B.** Lig-37 mediates formation of shorter coding joints. Recombined plasmid DNA was extracted from Amp/Cam-resistant colonies and subjected to DNA sequencing. Sequencing results were analyzed, and total joint length was calculated and plotted. The length of coding joints formed with Lig-IV versus Lig-37 differed significantly in the number of nucleotides (Nt) at the junction, with the average joint length of Lig-37 shorter than that of Lig-IV. **C** and **D**. Lig-37 supports formation of coding joints with increased nucleotide deletion and decreased nucleotide insertion. For Fig 2B–2D, vertical scatter plots depicts individual coding joint lengths, nucleotide deleted, and nucleotides inserted respectively. For each data set, a two-tailed student’s t-test was performed and the p-values indicate a significant change between the values obtained in junctions isolated from Lig-IV versus Lig-37 transfected cells. White arrows within the graph highlight the mean value for each set of data points.

To determine whether Lig-36, Lig-37, and/or Lig-43 could support V(D)J recombination, we transfected the expression vectors encoding these ligases, along with the coding joint substrate pGG51, into the Lig-IV-deficient cell line N114P2 [11]. The amount of DNA transfected for each expression vector was pre-determined to ensure comparable levels of protein expression, and it was the same as shown for Fig 1B. 48 hours after transfection, plasmid DNA was recovered and coding joint formation was measured using the colony formation assay as described in Materials and Methods [32, 33]. Only Lig-37, containing the DBD and C-terminal region of Lig-IV and the NTD and OBD from Lig-I complemented coding joint formation (Fig 2A). Lig-37 showed, in average, a 25% of coding joint formation activity when compared to Lig-IV. This result suggests that Lig-IV DBD and C-terminal region contain the required information to allow participation of Lig-IV in V(D)J recombination. It also indicates that the NTD and OBD domains of Lig-I can substitute for the NTD and OBD domains of Lig-IV and are able to contribute to the function of the chimeric protein Lig-37. This finding might be in part explained by the observation that among the three ligases present in mammalian cells, the NTD and OBD domains are the most similar [16]. Lig-I normally catalyzes ligation of a single strand nicks [17, 38]. In the context of chimeric Lig-37, Lig-I NTD and OBD are contributing to the ligation of double stranded breaks generated by the Rag recombinase, demonstrating that these domains are interchangeable. Interestingly, DNA Ligase III (Lig-III) was recently shown to contribute to the repair of DNA double stranded ends in the context of a catalytically inactive Lig-IV and in 293T cells [39]. It is unlikely that Lig-III plays any role in the activity that we observe with Lig-37 in N114P2 human pre-B cells given that catalytically inactive Lig-IV mutants were inactive in signal and coding joint formation when expressed and analyzed in N114P2 cells [40].

To determine whether coding joints generated by Lig-37 were comparable to the coding joints produced by wild type Lig-IV, coding junctions from recombinant colonies were sequenced. Coding joints generated in cells complemented with Lig-37 were shorter than those produced in cells complemented with wild type Lig-IV (Fig 2B). In three independent experiments, we observed a decrease in coding joint length in cells complemented with Lig-37. In average, within the three experiments, decrease in coding joint length ranged from 3 to 8 bp pair shorter. Further analysis of the sequences indicated that the shorter coding joints could be explained by an increase in nucleotide deletions, and a decrease in insertions observed in coding joints recovered from cells transfected with Lig-37 (Fig 2C and 2D, and Tables 1.1 and 1.2 in S1 Table). We cannot rule out that the decrease in N nucleotide addition could be a consequence of enhanced nucleolytic activity at the coding end; N nucleotides could be added and subsequently deleted and not present in the final coding joints. The changes observed in junctional diversity, with increased deletion and decreased N nucleotide addition suggest significant differences in the functionality of Lig-37 when compared to Lig-IV. Changes in the interaction and/or the recruitment of processing factors, and/or overall alterations in the utilization of microhomology during processing and ligation might explain some of the differences observed in the coding joints generated by Lig-37 versus those generated by Lig-IV. In addition, some of these changes could potentially include decreased activity of Lig-37 in cells, which could favor deletions and microhomology use at the coding ends. However, in the *in vitro* ligation assay presented in Fig 1C, Lig-IV and Lig-37 show low but comparable levels of activity.

### Overexpression of Lig-37 in mouse pro-B cells induces changes in junctional diversity of endogenous chromosomal substrates

**Fig 3:**
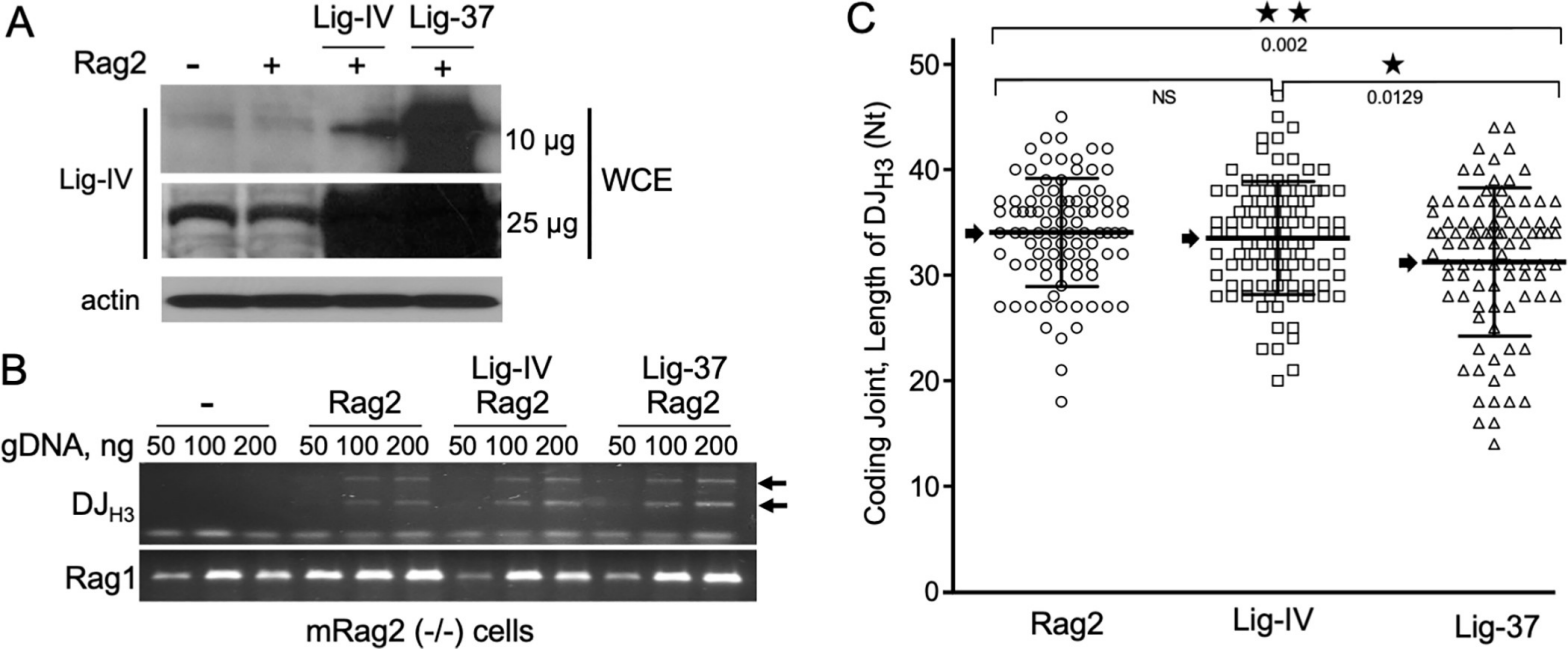
Formation of shorter endogenous coding joints in mouse pro-B cells overexpressing Lig-37. **A.** Overexpression of human Lig-IV and Lig-37 in mouse pro-B cells undergoing endogenous recombination. Rag2^-/-^ mouse pro-B cells expressing human Lig-IV or Lig-37 as well as control cells that express only endogenous ligase were transduced with Rag2 to induce recombination. At day 8 post transduction with Rag2 expressing virus, ligase expression was analyzed by western blot using an antibody generated against the DBD of Lig-IV [23]. The two upper panels show immunoblots containing 10 and 25 μg of cell lysate and probed with anti-Lig-IV DBD antibodies. In the bottom panel, 5 μg of cell lysate was used to probe with anti-actin antibodies as control. **B.** PCR amplification of endogenously recombined DJ_H3_ locus. Analysis of V(D)J recombination activity via PCR amplification of the endogenous DJ_H3_ locus is shown in the upper panel. As per A, gDNA for PCR analysis was collected 8 days post transduction with Rag2 expressing virus. PCR products were resolved on an agarose gel and stained with Ethidium bromide. Amplification of the *Rag1* gene was used as PCR control (bottom panel). **C.** Comparative length of endogenous DJ_H3_ coding joints. Amplified PCR product generated as indicated in B were cloned, sequenced and analyzed for coding joint length. Vertical scatter plot depicts each individual coding joint length. A two-tailed student’s t-test was performed, and the *p* value indicates a significant change in (**) coding joint length with Lig-37. Three independent experiments showed similar results. Black arrows within the graph highlight the mean value for each set of data points.

To investigate whether Lig-37 could contribute to coding joint formation of the endogenous immunoglobulin gene segments, an overexpression approach was used; Lig-IV and Lig-37 were overexpressed in the Rag2-/- mouse pro-B cells, which express endogenous mouse Lig-IV. Rag2-deficient mouse pro-B cells [28] were first transduced with vectors expressing Lig-37 or control human Lig-IV. Stable pools of Lig-37 and control Lig-IV expressing cells were further transduced with Rag2 expressing virus, selected and expanded for 8 days. Cells harvested 8 days after Rag2 transduction showed high levels of expression of Lig-IV and Lig-37 (Fig 3A; last two lanes). Endogenous Lig-IV was only observed by western blot analysis when higher levels of protein were loaded into the gel and the gel was overexposed (Fig 3A; first two lanes, 25 μg). All cells transduced with the Rag2 virus showed evidence of V(D)J recombination as determined by PCR amplification of the DJ_H3_ gene segment (Fig 3B). Recombination levels of the DJ_H3_ segments were comparable between cells expressing endogenous Lig-IV and those over expressing human Lig-IV or Lig-37. Sequence analysis of the coding junctions generated upon DJ_H3_ rearrangement showed a decrease in the length of coding joints of cells overexpressing Lig-37 (Fig 3C). This decrease was not observed upon overexpression of the control human Lig-IV. Together, results presented in Fig 3 suggest that Lig-37 is able to participate in recombination of the endogenous locus. This data further supports our conclusion that Lig-IV DBD and C-terminal region contain the required information to allow participation of Lig-IV in V(D)J recombination, and potentially facilitate the recruitment of the NHEJ proteins to the V(D)J repair process. Chimeric proteins like Lig-37 might constitute a valuable tool by offering approaches that might change the bias of the antigen receptor repertoire and potentially provide novel specificities with shorter CDR3 regions. Lig-37 might also help in further understanding the mechanisms of junctional diversity and the specific requirements of the NHEJ pathway in V(D)J recombination.

### Lig-37 supports signal joint formation

**Fig 4:**
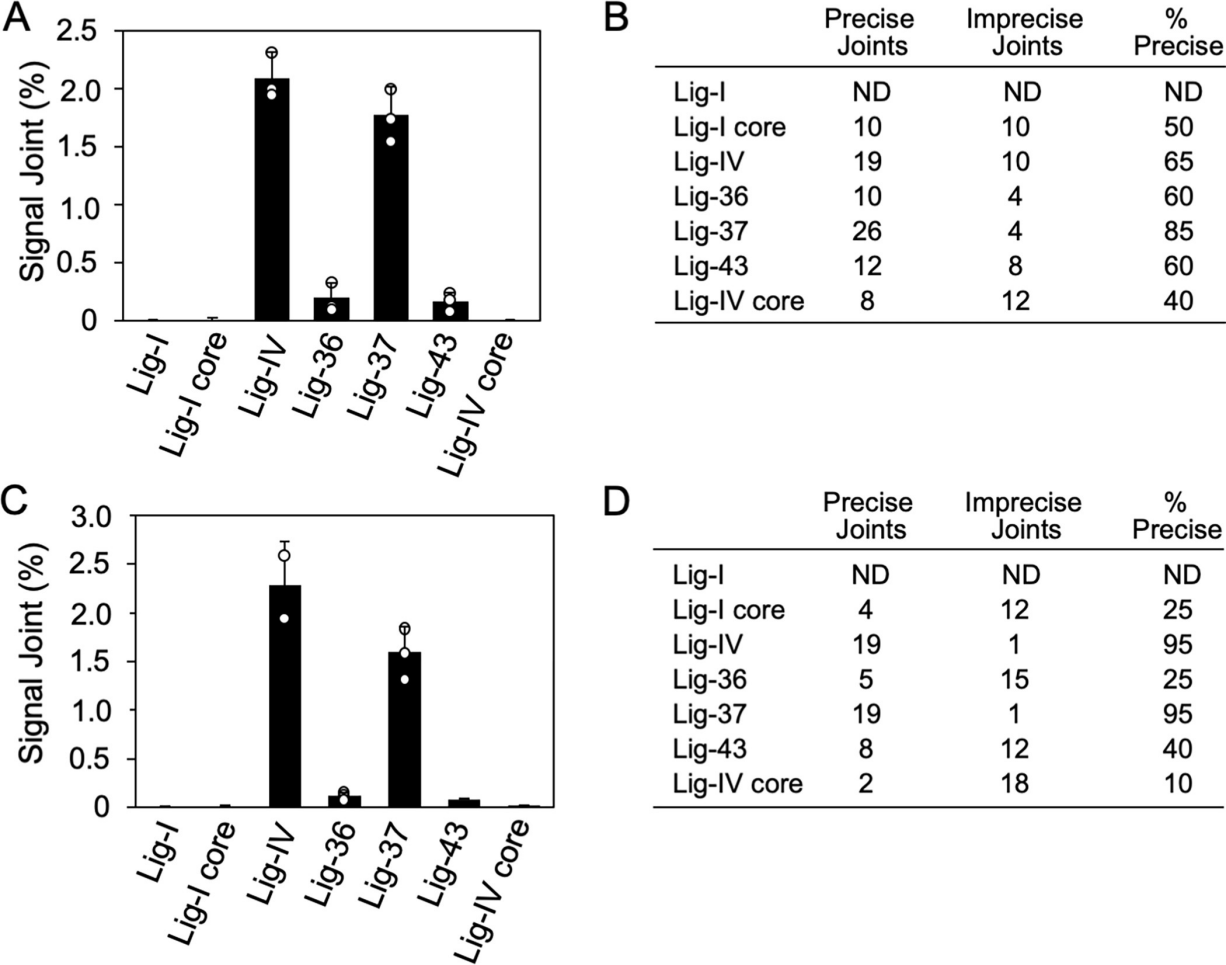
Lig-37 supports signal joint formation. **A.** Lig-37 supports signal joint formation in Lig-IV-deficient human pre-B cells. Lig-IV-deficient N114P2 human pre-B cells were transiently transfected with Flag-tagged chimeric ligase expression vectors or controls, along with the signal joint (SJ) substrate, pGG49. Two days post transfection, plasmid DNA was extracted from the cells and digested with DpnI, followed by transformation. SJ formation was determined by a colony formation assay as described [32]. Percent of SJ formation is shown in the y-axis. Error bars are from the evaluation of three biological replicates. The individual data points from the biological replicates are depicted within the graph. **B.** and **D.** Percent of precise signal joints generated by the chimeric ligases. For the SJ analysis performed in B and D, two independent experiments were done. The results from both experiments were combined and are presented in the tables. **C.** Evaluation of SJ formation in Lig-IV MEFs. Similar to the experiment presented in A, however, Lig-IV deficient MEFs were transfected with the murine SJ recombination substrate pJH200 [34]. Error bars are from the evaluation of triplicates performed in this experiment, except for the Lig-IV data point that shows the average of two biological replicates. Data presented in A and C is representative of two independent experiments.

Signal and coding joints represent the final products of V(D)J recombination. To determine whether the chimeric ligases could support the generation of signal joints, signal joint substrates were co-transfected with vectors expressing Lig-36, Lig-37, Lig-43 or controls Lig-I, Lig-I core as well as Lig-IV and Lig-IV core into Lig-IV-deficient cells. Recombination substrate along with recombined products were isolated from transfected cells and analyzed for signal joint formation via the colony formation assay as described in Material and Methods [32]. Results presented in Fig 4A and 4C indicate that Lig-37 was active in signal joint formation. Lig-36 and Lig-43 showed a low level of signal joint formation. For signal joint formation, similar results were obtained in human N114P2 cells and in Lig-IV-deficient MEFs. In both, Lig-IV-deficient cellular systems, expression of Lig-37 showed over 70 percent of activity in signal joint formation when compared to Lig-IV. And despite generating coding joints with increased deletions (Figs 2 and 3), the signal joints isolated from cells expressing Lig-37 were more than 80 percent precise as determined by ApaL1 digestion (Fig 4B and 4D).

In summary, Lig-37 that contains the DBD and C-terminal region of DNA Lig-IV flanking the NTD and OBD of DNA Lig-I was very efficient at complementing signal joint formation, showing over 70 percent of activity when compared to Lig-IV. Signal joints generated by Lig-37 were mostly precise (85 percent of precise joints in N114P2 cells and 95 percent of precise signal joints in Lig-IV deficient MEFs). In contrast, while Lig-37 could support on average 25 percent of coding joint formation activity when compared to Lig-IV, the coding joints generated in cells expressing Lig-37 were shorter and showed a decreased level of N nucleotide addition. This might reflect changes in the structure of Lig-37 when compared to Lig-IV. Lig-I NTD and OBD present in Lig-37 might provide decreased protection to the DNA double-stranded ends, and/or change the use of microhomology, thus leading to enhanced deletions. The presence of the NTD and OBD of Lig-I in Lig-37 could also lead to decreased recruitment of polymerases such as TdT, resulting in lower levels of N nucleotide addition. It is also possible that the Lig-IV NTD and OBD contains information that normally contributes to regulating coding end processing. While the analysis that we have done cannot provide definitive answers for the observed changes in coding end processing, our data highlight the critical role that the DBD and C-terminal region of Lig-IV play in allowing participation of Lig-IV in V(D)J recombination.

## Supporting information

S1 Table Sequence analysis of coding joints from recombined pGG51. As in Fig 2, Lig-IV deficient cells (N114P2) were transfected with coding joint substrate pGG51 along with control Lig-IV or with Lig-37. Recombined pGG51 was purified from Amp/Cam colonies and sequenced. On both tables, the top sequences correspond to a non-modified coding joint. Dashed lines represent deleted nucleotides. Sequences in the middle panel of Tables 1.1 and 1.2 represent insertions that could be N or P nucleotides.
(TIFF)

S1 Raw images (PDF)
