# Supplementary figures and images for: The DNA binding domain and the C-terminal region of DNA Ligase IV specify its role in V(D)J recombination
Source: PLoS One. 2023 Feb 24;18(2):e0282236. doi: 10.1371/journal.pone.0282236 (PMC9956705; doi:10.1371/journal.pone.0282236)

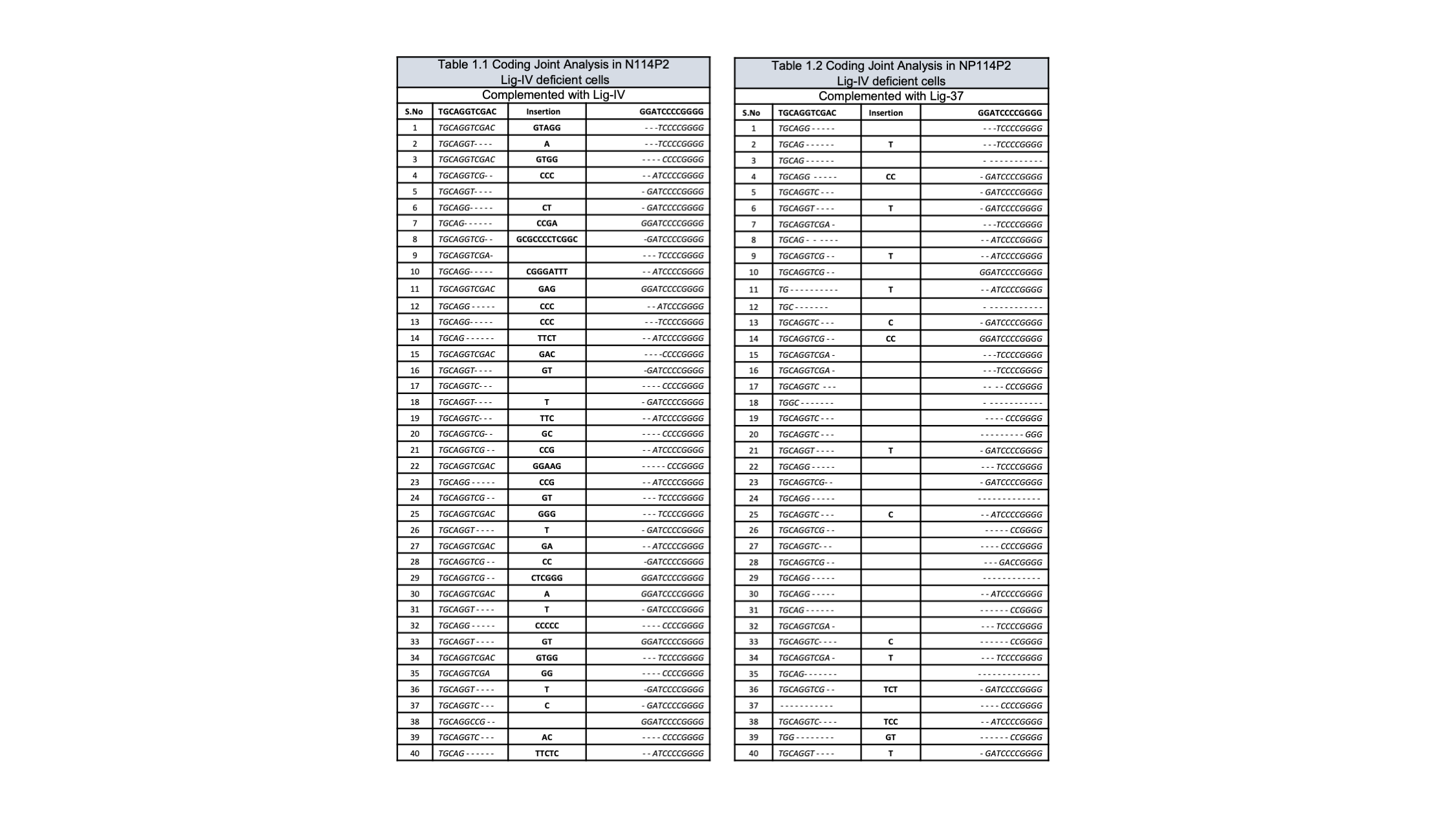

Supplement: S1 Table — As in Fig 2, Lig-IV deficient cells (N114P2) were transfected with coding joint substrate pGG51 along with control Lig-IV or with Lig-37. Recombined pGG51 was purified from Amp/Cam colonies and sequenced. On both tables, the top sequences correspond to a non-modified coding joint. Dashed lines represent deleted nucleotides. Sequences in the middle panel of Tables 1.1 and 1.2 represent insertions that could be N or P nucleotides. (TIFF) [file pone.0282236.s001.tiff]

Fig 1B

B

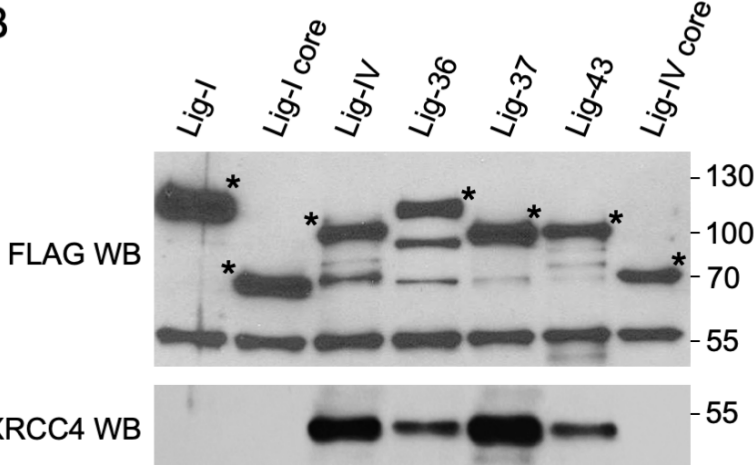

FLAG WB

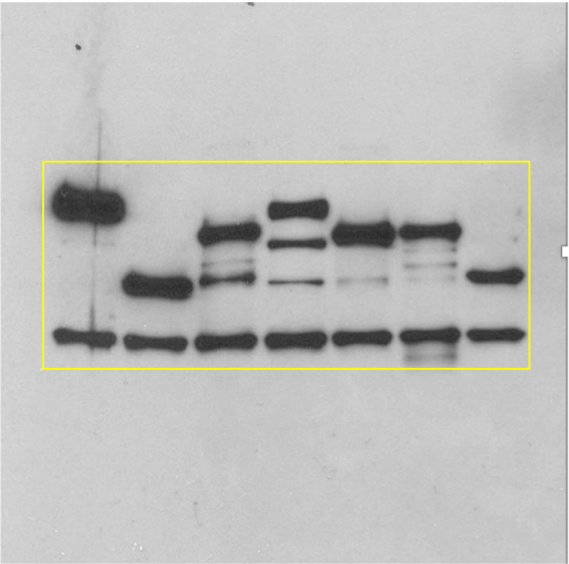

XRCC4 WB

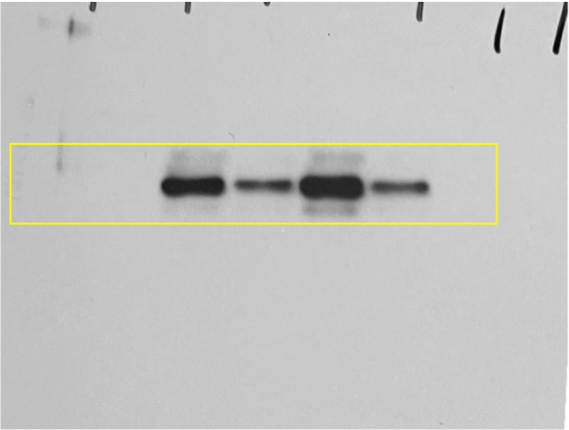

Fig 1C

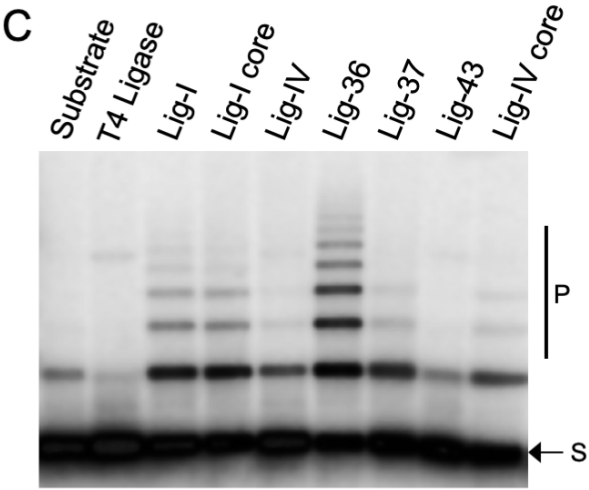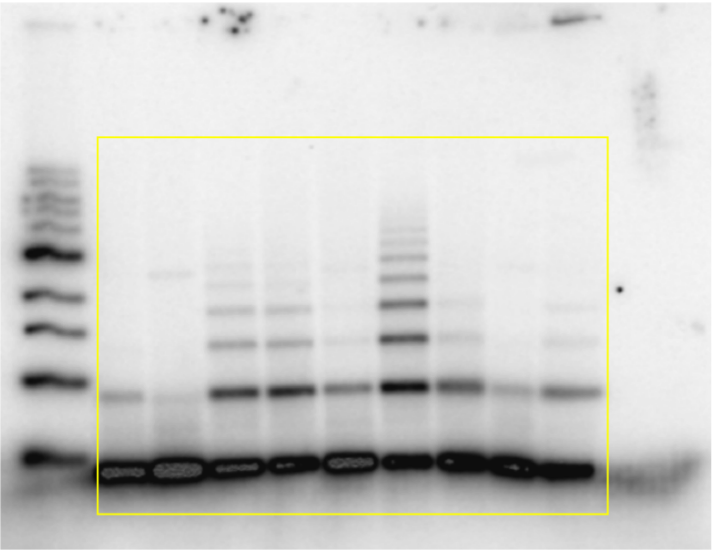

Fig 3A

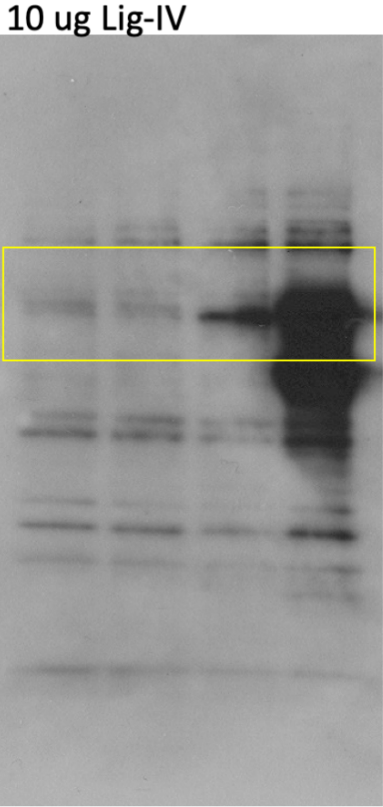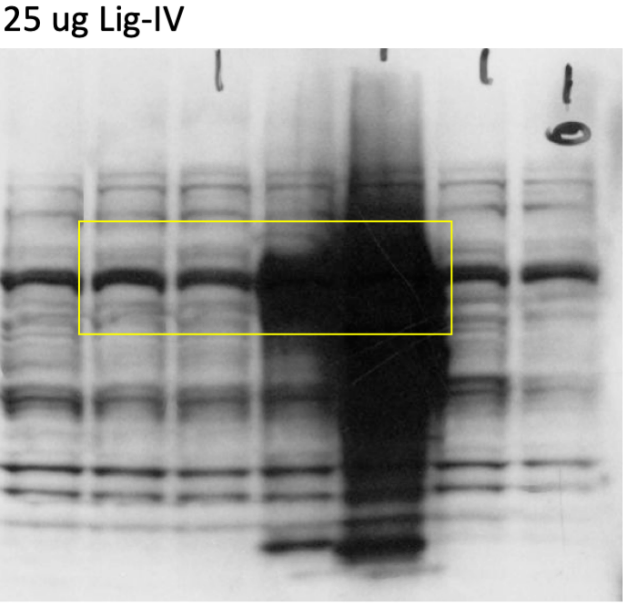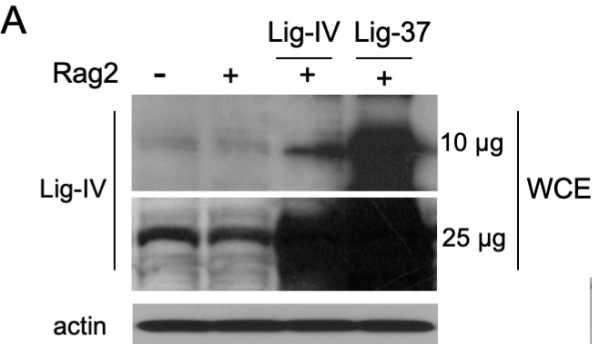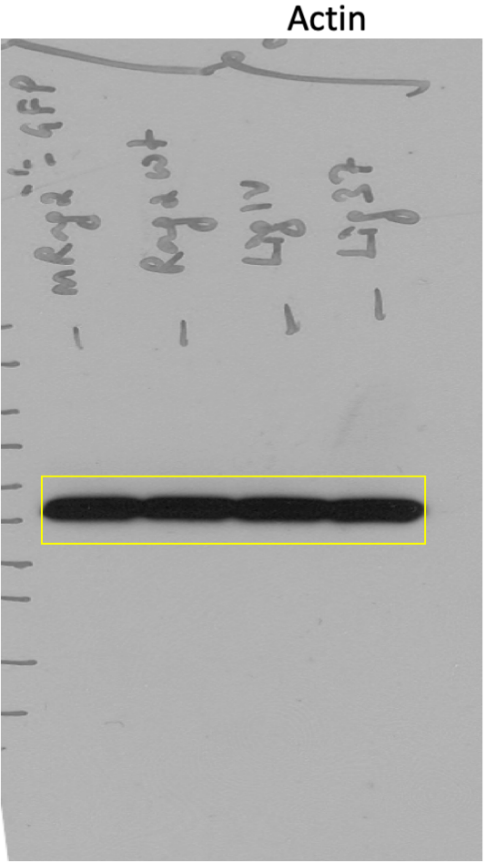

Fig 3B

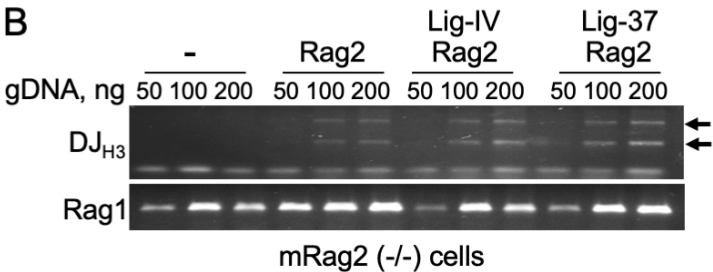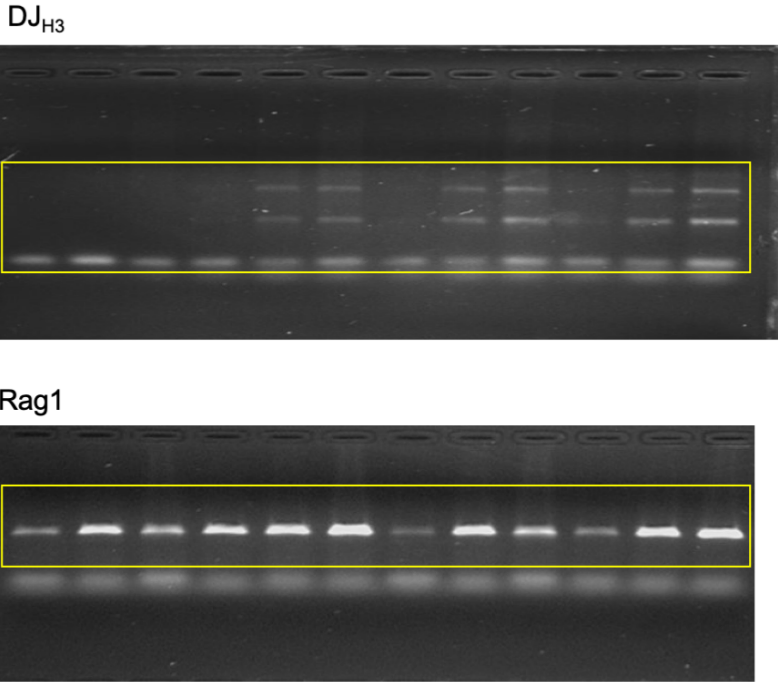

Supplement: S1 Raw images — (PDF) [file pone.0282236.s002.pdf]
